# Neural Correlates of Experience-Induced Deficits in Learned Vocal Communication

**DOI:** 10.1371/journal.pone.0014347

**Published:** 2010-12-16

**Authors:** Isabelle George, Sandrine Alcaix, Laurence Henry, Jean-Pierre Richard, Hugo Cousillas, Martine Hausberger

**Affiliations:** UMR6552-Ethologie Animale et Humaine, Université Rennes1-CNRS, Rennes, France; Claremont Colleges, United States of America

## Abstract

Songbirds are one of the few vertebrate groups (including humans) that evolved the ability to learn vocalizations. During song learning, social interactions with adult models are crucial and young songbirds raised without direct contacts with adults typically produce abnormal songs showing phonological and syntactical deficits. This raises the question of what functional representation of their vocalizations such deprived animals develop. Here we show that young starlings that we raised without any direct contact with adults not only failed to differentiate starlings' typical song classes in their vocalizations but also failed to develop differential neural responses to these songs. These deficits appear to be linked to a failure to acquire songs' functions and may provide a model for abnormal development of communicative skills, including speech.

## Introduction

Birdsong, like speech, is a learned behaviour whose development critically depends on social interactions [1], [2]. In highly social songbirds such as starlings, the lack of direct social contacts with adults severely impacts song development [3]–[6].

**Figure 1:**
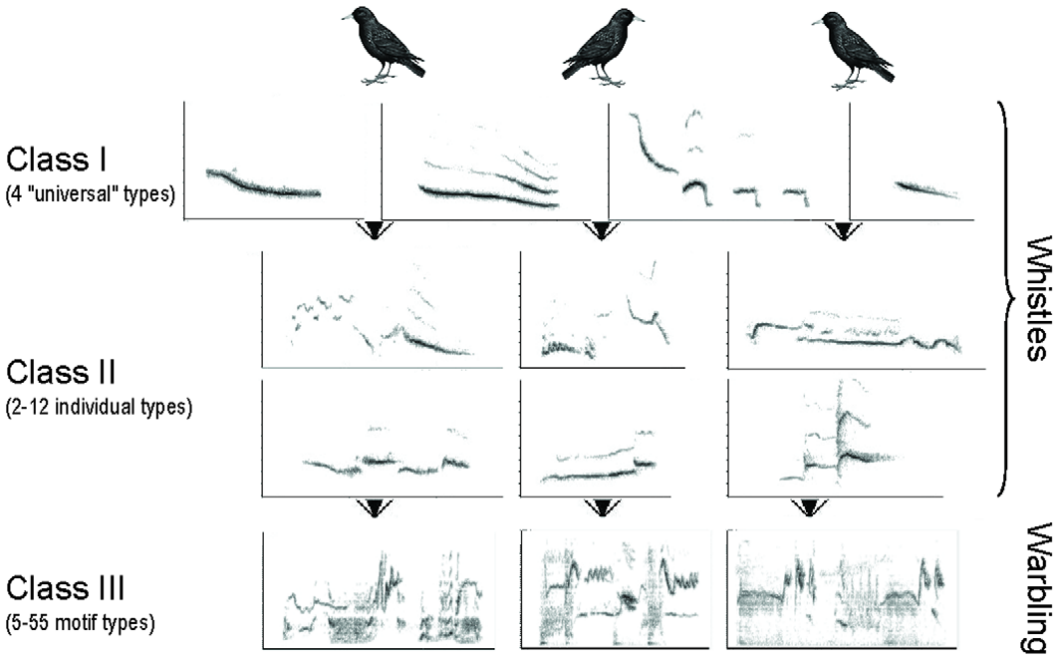
Main song classes of male European starlings, based on field observations of more than 300 birds. Class-I whistles (top row) are present in the repertoire of all or most males, with the same characteristics and variation range. They are subdivided into 4 main types or themes (from left to right): inflection theme, theme with harmonics, rhythmic theme and simple theme. Class-II songs (middle rows) are individual whistles that characterize one bird in its colony. Bottom row shows parts of the class-III song (also called warbling) including 1-2 motifs (which are the basic acoustic and perceptual units of warbling [5], [16]). Modified from Hausberger [7].

Starlings are songbirds with a particularly sophisticated vocal communication system that has been well described (e.g. [7], [8]). Male starlings sing three classes of songs (see Fig. 1) that differ not only by their structure [9], [10] but also by their pattern of acquisition during song learning [3]–[5] and by their context of emission [11]–[15]. Class-I and –II songs are short, simple and loud whistles that are usually produced in a discontinuous way, with typical intervals of 1–8 s, and class-III songs are long, complex and soft songs, also called warbling, that are produced in long, continuous sequences of motifs (fixed, repeatable combinations of notes that are the basic acoustic and perceptual units of warbling [5], [16]) with no silent interval longer than 0.5 s [7], [8].

Whistles show different levels of variation that allow not only species and population recognition (class-I songs) but also individual recognition (class-II songs) [7]. They are thought to be important for social relationships because song matching between neighbours occurs only with the whistled songs [8]. Class-I songs are subdivided into 4 main types or themes (see Fig. 1) that are sung by all male starlings and that are used in species and population recognition in the wild [11], [17]. They are mainly used in long-distance vocal interactions between males [11], and they are hardly produced by captive birds, either wild-caught or hand-raised [3], [4], [12]. In addition to these “universal” class-I songs, each and every starling has a unique repertoire of 2-12 class-II whistles (see Fig.1 for examples) that are used in individual recognition, especially between same-sex social partners [18]. In the wild, class-II whistles characterize one bird in the colony [19] and they usually do not evoke vocal interactions [20]. In captivity however, they can be shared by 2-3 same-sex social partners and they evoke vocal interactions, in both males and females [12], [18]. They are often produced in series just before the start of a warbling sequence and they are thus thought to be a way to attract the attention of a receiver from a distance before starting the quieter warbling [20].

**Figure 2:**
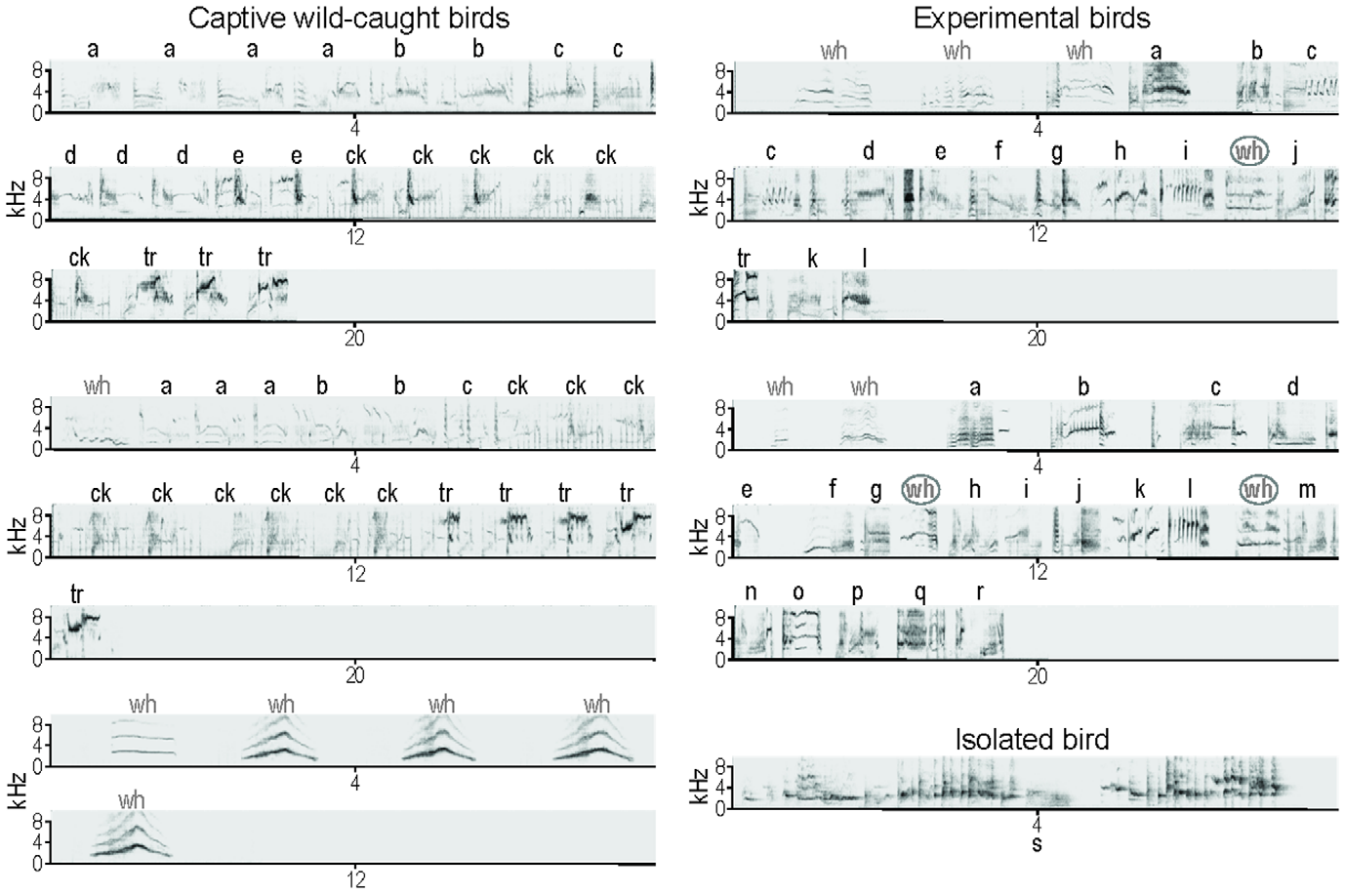
Experimental birds showed deficits in their vocalizations. Whereas captive wild-caught birds (left panel) usually produce class-II whistles (wh) either independently (bottom rows) or just before long, continuous class-III, warbling sequences (top and middle rows) showing a clear tripartite organisation: individual-specific variable motifs (a–e) that are repeated several times and followed by species-specific click motifs (ck) and high pitch trills (tr) that end the sequence, experimental birds (right panel, top and middle rows) produced short and desultory warbling sequences, with few repetitions of each class-III motif (a–r), long intervals between motifs and no clicks, and they kept switching back and forth between class-II whistles (**wh**) and class-III, warbling motifs. However, these song sequences clearly differed from those produced by isolated birds (right panel, bottom row). Letters in grey indicate parts that belong to class II and letters in black indicate parts that belong to class III. Bold letters indicate class-II whistles that are found within warbling sequences. Songs from captive wild-caught birds' and from an isolated bird come from song libraries recorded in our laboratory (unpublished data).

Finally, class-III songs are mainly composed of individual-specific motifs but also of some motifs that are common to all male starlings. All warbling sequences show the same general tripartite organization (see Fig.2 for an example): 1) variable motifs that are repeated several times, 2) click motifs and 3) high-pitched trills (variable motifs, click motifs and high-pitched trills all belong to class III) [5], [8], [19], [21]–[23]. Whereas variable motifs are highly individual in the wild (in captivity, some motifs may be shared by social partners), click motifs and high-pitched trills are found in the repertoire of all male starlings. Warbling is involved in short-distance communication, especially between males and females, and it is thought to play a role in mate choice [24], [25]. Its continuous structure does not allow distant, alternating vocal interactions.

Data on starlings show that young birds raised without any adult or without any direct contact with adults develop atypical songs showing both phonological and syntactical abnormalities. Thus, in socially-deprived starlings, some of the song classes described above can be absent [3]–[5], [26]. Starlings raised in captivity for example do not produce class-I songs [12] and young starlings raised in pair do not produce class-II whistles and some class-III motifs [3]. This raises the question of what functional representation of their vocalizations such deprived animals develop and what are the neural consequences of such abnormal development.

One plausible site for sensory representation of birdsong in the avian brain is the caudo-medial nidopallium (NCM), which is analogous to the mammalian secondary auditory cortex [27]. Based on electrophysiological responses [28]–[31] or on the expression of an immediate early gene (IEG – ZENK) [32]–[34], NCM auditory responses have been shown to be the strongest for conspecific songs, followed by heterospecific songs and non-song acoustic signals, and they are known to show a rapid and long-lasting habituation effect that is song-specific. NCM neurons thus appear to be able to discriminate between different conspecific songs, a property that is required for perceptual song discrimination. Moreover, NCM neurons show responses that parallel categorical preferences of male zebra finches for the long calls of females over those of males (as revealed by quantification of zebra finch calling in response to long call playbacks; [35]). It has been suggested that the mechanisms that account for the differences in the response magnitude to various auditory stimuli could be related to processes that support discrimination and categorical perception [36] and NCM has thus been proposed as a functional pattern recognition system [37]. More recently, we have observed differential responses to the ethologically-defined starlings' classes of songs in the NCM of adult male starlings [38], which suggests that this non-primary, associative auditory area could well be the place for sorting sounds into functional categories in the songbird brain.

The complex response properties of NCM have been shown to change with experience and to be involved in developmental vocal learning (e.g. [39]). Thus, stronger responses have been observed for songs associated with reinforcement [16]. Moreover, Fos responses appear to be correlated with the number of elements copied from a tutor [40], [41]. Finally, according to Chew et al. [28], NCM would play a role in the establishment of long-lasting memories for conspecific vocal signals. In the present study, we hypothesized that the absence of direct contact with adults during development may alter not only song production (as already demonstrated [3]–[6], [42]) but also the ability to categorize functional classes of songs (as reflected by differential responses to these songs, like those we observed in wild-caught adult male starlings [38]).

## Results and Discussion

In order to interfere with song development, we raised male starlings that we deprived of any direct contact with adults. Ten young male starlings were collected from the nest and hand-reared until they reached independence. They were then placed in a large outdoor aviary until the age of 5–6 months, before full song began to emerge (at the age of 6–9 months [5]). In this aviary, they could hear and see wild birds (including starlings) living on the campus but they could not directly interact with them. After this period, they were placed in an indoor aviary and, from this date, they were housed in the laboratory, with no adult starlings in the vicinity, until song and electrophysiological recordings were made. This protocol ensured that, although our birds had a chance to hear species-specific song models during their first months of life, they had no chance to interact with adults before full song began to emerge, and thus to use their songs in a normal communication network.

As expected, recordings of the vocalizations produced by these experimental birds at the adult age of 2 years revealed that they had developed atypical songs characteristic of birds lacking experience with adults [3], [4], [26]. All experimental birds sang, yet they never produced class-I songs (which is usual in captive birds, either wild-caught or hand-raised [3], [4], [12]). They did however produce class-II and class-III songs showing species-typical acoustic morphology (as assessed by visual inspection of sonograms by 2 experienced and 1 naïve observers; see Materials and Methods and Fig. 2), but these song classes were not found in their usual place in the song sequence. Experimental birds produced short and desultory warbling sequences, with few repetitions of each motif, long intervals between motifs and no clicks, and they kept switching back and forth between class-II and –III songs. Thus, class-II whistles were found within warbling sequences, with short silent intervals, while intervals between class-III, warbling motifs were longer than usual (see Fig. 2). It therefore appeared that class-II and -III songs were not differentiated in the experimental birds' vocalizations. However, these vocalizations clearly differed from those of purely isolated birds that never heard normal songs (see Fig. 2), indicating that our experimental birds did hear normal songs during their stay in an outdoor aviary and that the lack of interactions with adults probably played a crucial role in the observed vocal disorders.

In order to assess the neural correlates of these disorders, the 10 experimental birds were subject to electrophysiological recordings throughout the NCM. As we said before, this non-primary, associative auditory area has been shown to differentially respond to the functional classes of starlings' songs [38]. We recorded the activity of 2186 NCM neuronal sites (mean±SEM = 219±16 sites/bird), while broadcasting artificial non-specific sounds (pure tones and white noise) and natural species-specific stimuli corresponding to the three classes of starlings' songs (classes I, II, and III; see ref. [7]). Recordings were made in both hemispheres but, since no difference between hemispheres was found, data of both hemispheres were pooled (see Materials and Methods).

**Figure 3:**
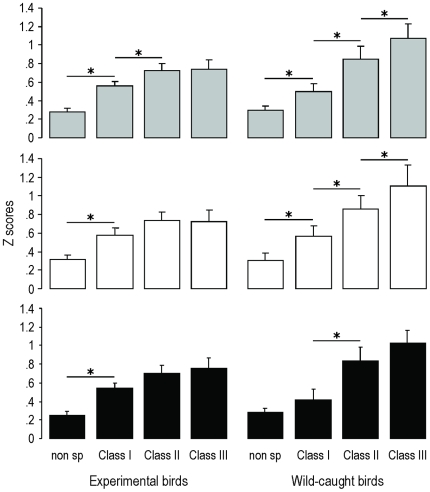
Experimental birds showed deficits not only in their vocalizations but also in their neural responses to sounds. Mean (+SEM) Z scores obtained for each class of stimuli in experimental (n = 10) and wild-caught (n = 6) birds (data for the latter come from George et al. [38]) showed that, whereas all pairwise comparisons were significant for wild-caught birds, no difference could be observed between class-II and class-III songs for experimental birds. Grey bars: pooled data of both hemispheres; white bars: data of the left hemisphere; black bars: data of the right hemisphere. *: p<0.05 according to post-hoc tests.

**Figure 4:**
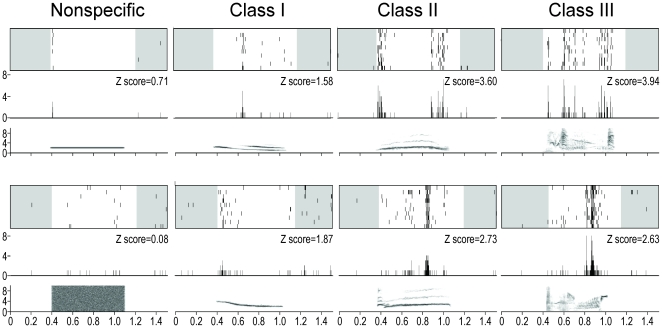
Examples of responses obtained in the NCM of 2 experimental birds. Neuronal activity is represented as raster plots corresponding to the ten repetitions of the stimulation (white areas indicate the time windows considered for auditory responses, that is from the beginning of the acoustic stimulus to 100 msec after its end), and as peri-stimulus time histograms (PSTHs) of the action potentials (that is, number of action potentials per 2-msec time bin) corresponding to the raster plots presented above. The sonograms of the acoustic stimuli (x axis: time in seconds; y axis: frequency in kHz) are presented below the PSTHs. All traces are time aligned.

Fourty seven percent of the recorded sites were responsive to at least one of the stimuli we used (mean±SEM = 46.8±3.6%). When only responsive sites (n = 1022; mean±SEM = 102±11 responsive sites/bird) were considered and further analysed, it appeared that the responses to the different classes of stimuli (as measured by normalized magnitude or Z scores) significantly differed (One-way repeated-measures ANOVA, F_3,27_ = 31.39, p<0.0001; Fig. 3 and 4). Responses to class-I songs were significantly higher than those to non-specific sounds (Tukey HSD, p = 0.0003), and responses to class-II and –III songs were significantly higher than those to class-I songs (Tukey HSD, p = 0.02 for Class I vs. Class II and p = 0.01 for class I vs. Class III). However, no difference could be observed between responses to class-II and class-III songs (Tukey HSD, p = 0.98; effect size, partial eta^2^ = 0.03). This was true not only across birds but also within 9 of the 10 experimental birds (Tukey HSD, p>0.19 in all cases; p = 0.03 for the remaining bird). It thus appeared that these two classes of songs were not differentiated at the neural level by the brain of the experimental birds. This contrasted with the clear differential NCM responses to these song classes that had been observed in wild-caught adult male starlings in a previous study [38] (see Fig. 3). Experimental birds therefore appeared to be unable to differentiate class-II and -III songs not only in their vocalizations but also in their neural responses to these songs, and this independently of the structural differences between these two types of songs. Interestingly, responses to class-II whistles were still significantly higher than those to class-I whistles, although the latter were very close to class-II whistles in structure. This suggests that the failure to develop differential responses to class-II and class-III songs is likely to reflect functional rather than structural deficits in our experimental birds' vocal behaviour. It could be argued that, given that class-I songs were all unfamiliar, and that class-II and –III stimuli also contained familiar songs, this could have influenced our results. However, since NCM responses have been shown to be higher for unfamiliar than for familiar stimuli [29], [39], such an influence would have led to higher levels of responsiveness for class-I songs than for class-II and –III songs, which is exactly the opposite of what we observed. Very recently, Thompson and Gentner [39] have shown that the overall strength of NCM responses to learned songs is inversely related to learned behavioural significance. This suggests that the higher, undifferentiated responses to class-II and –III songs that we observed in our birds could reflect a failure to learn the behavioural significance of these songs, indicating that this learning process requires direct, close interactions with adult models.

Our results show that the development of differential responses to starlings' functional classes of songs in NCM, such as those we observed in wild-caught adult males [38], is experience-dependent: young male starlings that we raised without any direct contact with adults not only failed to differentiate song classes in their vocalizations but also failed to develop differential neural responses to these songs.

Although the syntactical organization of the song was affected in our group of experimental starlings, the phonological structure of song units did not differ from those produced by wild-caught birds (see Fig. 2), suggesting that NCM might be involved in categorizing songs, not only depending on their acoustic structure, but also depending on their correct use in syntactical context. Moreover, the fact that song classes showing species-typical acoustic morphology were not found in their usual place in the experimental birds' song sequences suggests that the observed deficit in neuronal responses is likely to be linked to a failure to acquire songs' functions and may provide a model for abnormal development of communicative skills, including speech. Indeed, since human speech and learned vocalizations in songbirds bear behavioural and neural parallels, songbirds provide a genuine model for investigating the basic principles of speech and its pathologies [43], [44]. Although, at this stage, it is difficult to think of any precise human pathology, especially as pathologies involving speech disorders are so numerous and varied, the deficits we observed are somehow reminiscent of what is called auditory agnosia, which is defined as the impaired capacity to recognize sounds despite adequate hearing (e.g. [45]), often associated with paraphasia (a serious communicative disorder where the selection of inappropriate words in sentences leads to a breakdown in understanding between speaker and hearer [46]) [47]. However, in most cases, the occurrence of auditory agnosia has been attributed to brain lesions [48].

Our study describes a novel form of experience-dependent plasticity in NCM and provides a unique example of convergence between vocal behaviour (here showing experience-induced deficits) and neural activity. Together with our study on wild-caught starlings [38], it points to NCM as a potential neural substrate for functional representation of learned communication signals whose development is crucially dependent on interactions with adult models. In birds, as well as humans, the presence of an adult conspecific listener not only guides vocal development by providing a model to copy but also provides vocal or non-vocal behavioural feedback about the content of developing vocalizations [2], [49], [50], and it appears here that adult influence is also crucial for young individuals to develop neural substrates for functional representation of their learned vocalizations. Given that songbirds are one of the few vertebrate groups (with humans) that evolved the ability to learn vocalizations, we believe that the present study improves our knowledge of the representation of sound significance in the brain, and that it will participate in understanding the role of social interactions (especially with adults) on not only the development of communication skills but also the development of the brain structures that underlie such skills.

## Materials and Methods

### Ethics Statement

The experiments were performed in France (licence no. 005283, issued by the departmental direction of veterinary services of Ille-et-Vilaine) in accordance with the European Communities Council Directive of 24 November 1986 (86/609/EEC).

### Experimental animals

Young starlings hatched in the wild in Rennes (France) were collected at 5–16 days of age and hand-reared as a group including birds from different broods, using commercial pellets mixed with water. After reaching independence at the age of 6 weeks, all subjects were placed in a large outdoor aviary for 4 months. They were then placed in an indoor aviary and, from this date, they were housed in the laboratory, separately from adult birds, until song and electrophysiological recordings were made (at the adult age of 2 years). In the laboratory, artificial light matching the natural photoperiod was provided.

At the start of the experiment, 10 male birds were placed in individual sound-proof chambers in order to record their song repertoire, and a stainless steel pin was then attached stereotaxically to the skull with dental cement, under halothane anaesthesia. The pin was located precisely with reference to the bifurcation of the sagittal sinus. Birds were given a 2-day rest after implantation. From this time, they were kept in individual cages with food and water ad libitum. During the experiments, the pin was used for fixation of the head and as a reference electrode.

Because of difficulties in obtaining and raising large numbers of male hatchlings, we could not replicate the experiment. It is therefore not known if our results could be replicated with another group of birds raised in the same conditions. However, comparisons with published data on other groups of starlings or pairs of young whose experience with adult song had been manipulated support the data on song production presented here [3]–[5].

### Acoustic stimulation

The vocalizations of each bird were recorded in a soundproof chamber until the whole song repertoire was established for every bird (that is until no new motif could be observed, which corresponded to about 1 hour of continuous song and took 3–6 weeks). Recordings were analyzed using a PC with sound analysis and synthesis software [51]. Sonograms were calculated with a FFT using a 256-point Hanning window and a 128-point step. Sampling frequency was 22 kHz and pixel size 87 Hz x 11.5 ms. Songs were classified in the three classes of starlings' songs [7] by visual inspection of sonograms by 2 experienced and 1 naïve observers who reached 98% in agreement. Although class-I and –II songs are close in structure, class-I whistles are characterized by key features that make their distinction easy from class-II songs (see Fig.1). Nevertheless, our birds did not produce class-I songs, which is usual in captive birds, either wild-caught or hand-raised [12]. They did however produce class-II and –III songs that were similar to those observed in wild-caught birds, even at the submotif level (see Fig. 2).

**Figure 5:**
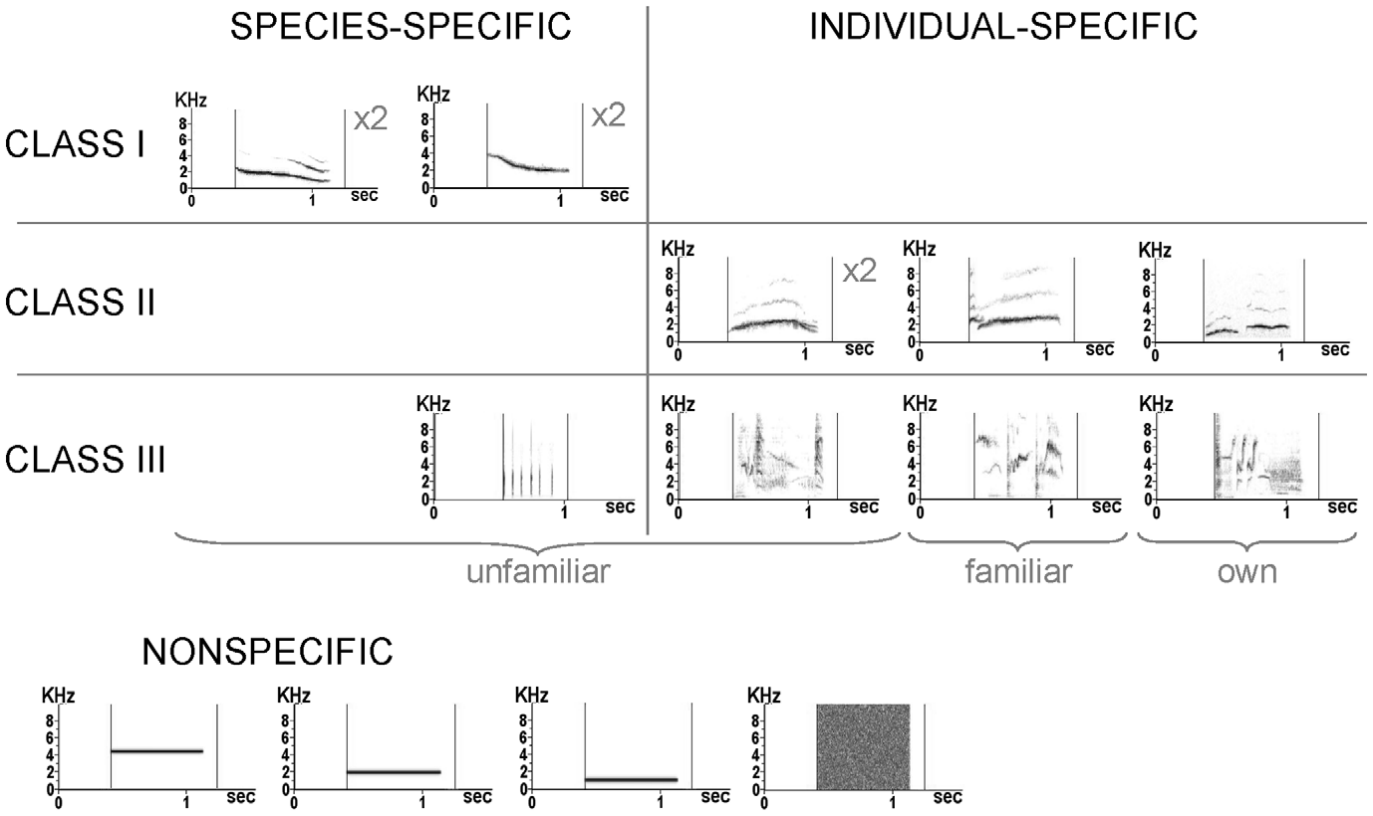
Stimuli that were used to test NCM responses in the experimental birds. Vertical lines on the sonograms indicate the time windows considered for auditory responses and correspond to the white areas of raster plots shown on Figure 4.

One class-II whistle and one class-III motif were chosen in the repertoire of each bird, and were used as familiar/own song stimuli. Class-II and class-III categories of stimuli were made of these familiar/own song stimuli and of unknown class-II and class-III songs of wild-caught birds (see below and Fig. 5). The bird's own class-II and class-III songs of one bird were used as familiar class-II and class-III stimuli for another bird, so that familiar and bird's own stimuli were counterbalanced across birds and class-II and class-III stimuli were identical across birds.

Sixteen acoustic stimuli were used (Fig. 5): 4 artificial non-specific stimuli (1-, 2- and 4-kHz pure tones and white noise), 4 class-I songs (all from unknown wild-caught birds), 4 class-II songs (2 unfamiliar and 2 familiar/own) and 4 class-III songs (2 unfamiliar and 2 familiar/own). The song stimuli were 400 to 817 ms long (mean±SD = 694.8±67.3 ms), and the artificial stimuli were 700 ms long, with 20 ms rise and fall times. The duration of the artificial stimuli was chosen in order to match the mean duration of the natural stimuli. The 16 stimuli were randomly interleaved into a single stimulus sequence that was repeated 10 times at each recording site. Within this sequence, different exemplars of the same stimulus class usually followed exemplars from different classes, thus ensuring that the order in which the stimuli were played to the birds (which was the same for every bird and every session) could not account for the observed pattern. The duration of the whole stimulus sequence was 25 s. The mean (±SD) interval between stimuli was 867.8±46.3 ms, with a minimum of 786 ms. Stimuli were delivered in an anechoic, soundproof chamber through a loudspeaker located 20 cm in front of the bird's head, with a peak sound pressure of 85 dB SPL at the bird's ears.

### Electrophysiological data collection and analysis

Neuronal activity during acoustic stimulation was recorded systematically throughout NCM, using the same approach as George et al. [52]. In brief, we used an array of 4 microelectrodes (2 in each hemisphere) made of tungsten wires insulated by epoxylite (FHC n°MX41XBWHC1), each spaced 1.11 mm apart in the longitudinal plane and 1.12 mm apart in the sagittal plane. Electrodes impedance was in the range of 3–6 MΩ. Recordings were performed during the breeding season (March-April) in an anechoic, soundproof chamber, in awake-restrained starlings, in one sagittal plane in each hemisphere, at 560 µm from the medial plane. Recordings in the left and right hemispheres were made simultaneously, at symmetrical locations. Each recording plane consisted of up to 10 penetrations systematically placed at regular intervals of about 220 µm in a rostrocaudal row, between 230–1090 and 2220–3080 µm from the bifurcation of the sagittal sinus. In order to stay within the limits of the NCM, only the most caudal penetrations (that is less than 2000 µm from the bifurcation of the sagittal sinus) were kept for analyses. Despite this precaution, we cannot rule out the possibility that a minor fraction of our data derives from recordings outside of NCM. If so, however, we might have expected to observe differences in the pattern of response within the sagittal plane, but no such differences were observed, and sites that responded to songs appeared to be homogeneously distributed throughout the recording plane. Only one session per day, lasting 3–4 h, was made, leading to 5–6 days of data collection for each bird. Between the recording sessions, birds went back to their cage, and a piece of plastic foam was placed over the skull opening in order to protect the brain. Birds were weighed before each recording session, and their weight remained stable over the whole data collection.

Neuronal activity was recorded systematically every 200 µm, dorso-ventrally along the path of an electrode penetration, independently of the presence or absence of responses to the stimuli we used, between 800–1800 and 4200–5200 µm below the surface of the brain. Spike arrival times were obtained by thresholding the extra-cellular recordings with a custom-made time- and level-window discriminator [52]. Single units or small multiunit clusters of 2-4 neurons were recorded in this manner. The data from both types of units were analyzed together, like in other studies [53], [54]. The computer that delivered the stimuli also recorded the times of action potentials and displayed on-line rasters of the spike data for the 4 electrodes simultaneously. At each recording site, spontaneous activity was measured during 1.55 sec before the presentation of the first stimulus of each sequence, which resulted in 10 samples of spontaneous activity (15.5 sec). Neuronal responsiveness was assessed as in George et al. [55] by comparing activity level (number of action potentials) during stimulation and spontaneous activity, using binomial tests. Only responsive neurons were further analyzed.

Z scores were used to assay the strength of neuronal responses (see Fig. 2). Z-scores are the difference between the firing rate during the stimulus and that during the background activity divided by the standard deviation of this difference quantity [56].

The mean values calculated for individual birds (n = 10) were used for statistical comparisons. Two-way repeated-measures ANOVAs (Statistica 8.0 for Windows, StatSoft Inc.) were performed to test for potential hemisphere and stimulus class effects. Since no difference between hemispheres was found (main effect of hemisphere: F_1,9_ = 0.19, p = 0.67; interaction: F_3,27_ = 0.36, p = 0.78), data of both hemispheres were pooled. These analyses were followed, when appropriate, by post-hoc comparisons with HSD Tukey tests (Statistica 8.0 for Windows, StatSoft Inc.). Unless otherwise indicated, data are presented as mean±standard error of the mean (SEM).
